# Factors associated with utilization of traditional Chinese medicine by white collar foreign workers living in Taiwan

**DOI:** 10.1186/1472-6963-9-10

**Published:** 2009-01-14

**Authors:** Maria Daly, Chen-Jei Tai, Chung-Yeh Deng, Li-Yin Chien

**Affiliations:** 1 Dublin Traveller Education and Development Group, Pavee Point, Dublin, Ireland; 2 Department of Traditional Chinese Medicine, Taipei Medical University Hospital, Taipei, Taiwan, Republic of China; 3 Department of Obstetrics and Gynaecology, Taipei Medical University, Taipei, Taiwan, Republic of China; 4 Institute of Hospital and Health Care Administration, National Yang-Ming University, Taipei, Taiwan, Republic of China; 5 Institute of Clinical and Community Health Nursing, National Yang-Ming University, Taipei, Taiwan, Republic of China

## Abstract

**Background:**

Traditional Chinese medicine (TCM) has remained an integral part of Chinese culture and society for thousands of years. In Taiwan TCM is a recognized element of its National Health Insurance Scheme. However, there is no knowledge about how TCM is accessed by foreign workers from a non-Asian cultural background. The objectives of this study were to investigate the prevalence and patterns of TCM use among non-Asian white-collar workers living in Taiwan, and examine factors likely to influence their use of TCM.

**Methods:**

This study applied a cross-sectional survey design. A total of 207 white-collar foreign workers of a non-Asian background currently holding National Health Insurance cards who had lived in Taiwan for 4 months or more participated in this study.

**Results:**

The prevalence of TCM use was 45%. The most frequently used therapies were traditional Chinese herbs/medicine and acupuncture. Factors indicating the likelihood of TCM usage were age 31–40 years, visit to an allopathic medical doctor in the last year, ability to read Chinese, having a friend or family member available to assist in the use of TCM, and access to information about TCM services available in Taiwan.

**Conclusion:**

Utilization of TCM by people of a non-Asian background living in Taiwan appears to be most influenced by enabling factors including language ability, access to information, and informal reference persons.

## Background

Taiwan, a modern Chinese country, has a National Health Insurance Scheme (NHIS) that provides coverage of general medical expenses to virtually all of its citizens and foreign workers [1]. Included in the NHIS are both modern allopathic medical care and traditional Chinese medicine (TCM). Although allopathic medicine is more predominant, over 3,700 licensed doctors of TCM practice in 2,500 hospitals and clinics around the country [1]. Patients are free to choose any health care provider registered under the NHIS for their care, without the need for approval by a gatekeeper.

The number of foreign residents in Taiwan has increased from 30,000 in 1991 to almost 293,000 in 2005 [2]. Much of this population is South East Asian but a significant number, around 15%, are professional/skilled or white collar workers from the United States, Australia, Canada and Europe [3]. There have been few studies of non-Asian foreign workers living in Taiwan and little is known about how this high level of TCM availability is accessed and used by this population. Previous studies about TCM utilization were mostly among people with a Chinese ethnic background [4-7]. Few have investigated TCM use by Western workers in an Asian country.

Anderson and Newman's model of health service utilization views an individual's use of health services as a function of predisposing factors (factors that exist prior to the health service utilization), enabling factors (the individual's ability to secure services), and need factors (the individual's illness level and health status) [8]. Their model provided a useful basis in a previous study investigating the use of complementary and alternative medicine among Chinese-American families in the United States [9]. The objectives of this study were to investigate the prevalence and patterns of TCM use among non-Asian white-collar workers living in Taiwan, and examine factors likely to influence utilization of TCM. Identification of factors indicating likelihood of TCM utilization was based on Anderson and Newman's model of health service utilization [8].

## Method

### Sampling

The study population was white collar foreign workers living in Taiwan for a period of at least 4 months. Non-Taiwanese nationals who identified themselves as having a Taiwanese or Chinese background were excluded from this study. Under present government identification non-Asian workers eligible to work in Taiwan are restricted to skilled or technical professionals with a minimum education level of a graduate degree or at least two years work experience in a skilled profession. Privacy laws prevented us from obtaining a sampling frame consisting of a list of names. Therefore, a non-random sampling method, network sampling, was applied, which was based on referrals from early members (key persons) already in the sample. Two procedures were used to recruit participants. In the first procedure, a key person was asked to distribute a letter of introduction and self-administered questionnaire to potential participants along with a stamped, addressed envelope. In this context key persons were longstanding members of the expatriate community in Taiwan and included staff at the Taipei Community Services Centre, International Community Radio Taiwan and other information points for interest and social groups, local clubs and sport leagues in and around Taipei. In the second procedure, a key person introduced the researcher to potential participants who, given the option, completed the questionnaire or were interviewed by the researcher. A total of 207 white collar foreign workers were included in this study. The response rate was 78.2%.

### Data collection

This study applied a cross-sectional survey design. A structured questionnaire was used to collect the data. The study participants either completed the questionnaire by themselves or were interviewed using the questionnaire, which took 12–15 minutes. An introductory letter and consent form along with a questionnaire was given to the potential study participants. A signed consent form was required for this study. The study was approved by the Institutional Review Board of National Yang-Ming University. The data was collected between January and April 2007.

### Measurement

The study variables were organized using Anderson and Newman's model of health service utilization [8]. Predisposing factors included socio-demographic characteristics such as age, sex, marital status, educational level, income, and nationality, along with health behavior (smoking and alcohol consumption). Enabling factors in workers living in a country where the language and culture is very different from their own considered the number of months/years they had lived in Taiwan, access to information about TCM services in Taiwan, ability to speak/read Taiwanese or Mandarin, and assistance from a friend or family member in using TCM. Need factors were physical pain in the past 2 weeks and use of hospital and allopathic health services in the last 12 months. TCM use was defined as having ever visited a practitioner and having application of traditional Chinese herbs/medicine, acupuncture and/or moxibustion, joint manipulation, acupressure and massage, dietary advice, or therapeutic exercise techniques such as qigong while living in Taiwan. Prior to distribution, the questionnaire was reviewed by three professors and piloted among seven English speaking foreign students in Taiwan to ensure that questions were appropriate.

### Data analysis

The data were analyzed using SPSS for Windows Version 13.0 (SPSS Inc, Chicago, IL, USA). The distribution and frequency of each category of variables were examined and chi-square tests were used to determine the association between TCM use and potentially related factors. Multivariate logistic regression using maximum likelihood estimations was then applied to examine factors associated with TCM use. We included all study variables (as described in the Measurement section) in the logistic regression model at the beginning. Variables that were not significantly associated with TCM use were then dropped from the logistic model. A p-value of less than 0.05 was considered statistically significant.

## Results

### Characteristics of study participants

Over 40% of the study participants (43.5%) were from Europe, especially the UK (27.5%), 37.2% were from North America (with 26.1% from the USA) and 3.4% were from South America. The distribution of nationalities in the sample was similar to that reported in the national statistics for foreigners in Taiwan. Approximately 74% of the study participants were males, which is also similar to national statistics.

Predisposing, enabling, and need factors are presented in Table 1. The mean age of the study participants was 35.6 years (range 21 to 62 years). About half (51.5%) had never married and 25% had between 1–4 children. The occupations represented were teachers or trainers 49.9%, skilled professionals (including engineers, technicians, journalists, pilots and health professionals) 26.6%, business and marketing executives 14.5%, managers 9.2%, government workers 2.4%, and others 1.4%. Almost 40% of participants smoked and over one-third were heavy drinkers.

**Table 1:** Characteristics of the study participants (N = 207; n1 = 94; n2 = 113)

Characteristics	All (% of n)	TCM use (% of n1)	Non-use (% of n2)	P
**Predisposing factors**				

Age				.006
21–30	35.7	30.4	40.7	
31–40	37.2	50.0	27.4	
41–50	18.4	15.2	21.2	
51–62	8.7	4.3	10.6	

Sex				.15
Male	73.9	69.1	77.9	
Female	26.1	30.9	22.1	

Currently married				.08
Yes	28.0	50.0	38.1	
No	72.0	50.0	61.9	

Education				.31
University	80.0	76.6	82.3	
Post-graduate	20.0	23.4	17.7	

Monthly income				.05
<= 100,000 NT	30.4	24.5	37.7	
>100,000 NT	69.6	75.5	62.3	

Smoker				.07
Yes	43.0	54.2	33.6	
No	57.0	45.7	66.4	

Alcohol consumed on usual night out or occasion when drinking^a^				.02
< 8 units	66.2	74.5	59.3	
>= 8 units	33.8	25.5	40.7	

**Enabling Factors**				

Family/friend available to assist in use of TCM				.001
Yes	63.8	76.6	53.1	
No	36.2	23.4	46.9	

Access to information of TCM services				.001
Very good/adequate	25.6	39.4	14.1	
Poor	74.4	60.6	85.8	

Ability to read Chinese				.001
<500 characters	81.6	71.3	90.3	
500 – 1000 characters	18.4	28.7	9.7	

**Need factors**				

Use of out-patient or emergency services in the last year				.21
Yes	41.1	54.3	62.8	
No	58.9	45.7	37.2	

Visit to an allopathic doctor about a health problem in the last year				.05
Yes	61.8	72.3	53.1	
No	38.2	27.7	46.9	

Physical pain in the last 2 weeks				.02
Yes	49.8	58.5	42.5	
No	50.2	41.5	57.5	

P value from χ^2 ^statistics.

^a^One unit of alcohol is described as a half a pint of beer, lager cider (3–4% alcohol by volume), single 25 ml measure of spirits (40% abv), and one and a half units as 125 ml (medium glass) of wine (12% abv)

Around 35% of participants reported themselves as able to speak a little Chinese or Taiwanese (up to 100 words) and 29% described their speaking ability at 500 – 1000 words. Most could not read any Chinese at all and only 18.4% could read 500 – 1000 Chinese characters. Just 9.2% of participants described access to information about TCM services in Taiwan as very good, and 16.4% as adequate. The most common length of time participants reported living in Taiwan was between 2 and 4 years.

Most participants had not been admitted to a hospital (91.8%) or used out-patient or emergency services in the previous year (58.9%). About 60% had visited an allopathic doctor about a health problem in the last 12 months. Most participants described their energy levels for everyday life as moderate or very good (37.2 and 51.2% respectively). Most participants reported no (49.8%) or little (35.7%) interference in their daily activities as a result of pain or discomfort.

### TCM utilization by participants

Ninety-four participants (45%) had utilized one or more of the TCM therapies included in this study. The most frequently utilized therapy was traditional Chinese herbs or medicines (42.6%), followed by acupuncture (37.2%) and massage (33.0%). More than one type of therapy was used by 40% of the TCM users. About half of TCM users (54%) reported more than one reason or condition for seeking a TCM practitioner. The most common reasons reported were muscular and joint problems 59.6%, lung and respiratory conditions 18.1%, and promotion of wellbeing or quality of life 16.0%. About 70% of TCM users rated their treatment as somewhat or very effective in addressing their condition, with 7% rating it as not at all effective (Table 2).

**Table 2:** Type, reason, and perceived effectiveness of TCM therapy among the TCM users (n = 94)

	n	%
Type of TCM used		
Traditional Chinese herbs or medicine	40	42.6
Acupuncture	35	37.2
Massage	31	33.0
Joint manipulation	13	13.8
Acupressure	11	11.7
Qigong	10	10.6
Dietary advice	8	8.5
Other therapeutic exercise	6	6.4
Moxibustion	5	5.3
Others	1	1.1

Reason for seeking TCM		
Muscular or joint problems	56	59.6
Lung or respiratory complaints	17	18.1
To promote wellbeing and quality of life	15	16.0
Stress	12	12.8
Fatigue or tiredness	11	11.7
Digestive problems	10	10.6
Chronic conditions or problems	9	9.6
Allergies or skin conditions	5	5.3
Prone to illness or low immunity	4	4.3
High blood pressure	3	3.2
Cholesterol condition	2	2.1
Cardiovascular disease	2	2.1
Kidney or bladder complaints	2	2.1
Reproductive or hormone complaints	1	1.1

Perceived effectiveness of treatment		
Very effective	24	25.5
Somewhat effective	42	44.7
Not really effective	21	22.3
Not effective at all	7	7.4

### Factors associated with TCM utilization

In bivariate analysis, eight variables were significantly associated with TCM use (age, income, alcohol use, family or friend available to assist in use of TCM, access to information on TCM services, ability to read Chinese, visit to an allopathic doctor about a health problem in the last year, and physical pain in the last 2 weeks; see Table 1). Of those, income, alcohol use, and recent physical pain were no longer associated with TCM use in multivariate logistic regression models. In the logistic model (Table 3), participants with an ability to read Chinese had the highest odds of TCM use (OR 3.14; 95% CI 1.33–7.40), followed by those who reported very good or adequate access to information about TCM services (OR 3.08; 95% CI 1.46–6.49). Those who had a family member or friend to assist in use of TCM were more likely to use TCM (OR 2.58; 95% CI 1.31–5.09). Participants who had visited an allopathic medical doctor about a health problem in the last year (OR 2.67; 95%CI 1.38–5.22) were more likely to be TCM users. Participants 31–40 years old were more likely to use TCM than those 21–30 years old (OR 2.30; 95% CI 1.20–4.42).

**Table 3:** Multivariate logistic regression model for TCM use among study participants

	Odds ratio	95% CI
Age (years)		
21–30	1.00	
31–40	2.30	1.20–4.42
41–50	0.91	0.37–2.24
51–62	0.61	0.16–2.34

Ability to read Chinese		
> = 500 characters	3.14	1.33–7.40
<500 characters	1.00	

Family and friends available to assist to use TCM		
Yes	2.58	1.31–5.09
None	1.00	

Information about TCM services		
Very good/adequate	3.08	1.46–6.49
Poor/don't know any information	1.00	

Visit to an allopathic medical doctor in last year		
Yes	2.67	1.38–5.22
No	1.00	

## Discussion

This study demonstrated that 45% of non-Asian white collar workers had utilized one or more types of the TCM therapies in Taiwan. There is an absence of knowledge concerning the use of traditional Chinese herbs/medicine and joint manipulation by non-Asian populations, but the rate was believed to be low. Acupuncture however, is becoming adopted as a form of health care in many countries, with utilization rates of 10 % reported in Australia, 9% in the UK, and 19% in Austria, [10-13]. Chen et al (2006) indicated a national TCM usage prevalence of 28.4% in the year 2001, and 62.5% of the population using TCM at least once during the 6-year period from 1996 to 2001 under Taiwan's NHIS [14]. The two most frequent types of TCM used in this study were traditional Chinese herbs/medicine and acupuncture, which is similar to patterns of use by the national population [14,15].

Participants used TCM predominantly for muscular and joint problems (59.6%). The other most common reasons or conditions stated were lung or respiratory complaints (18.1%); to promote wellness and quality of life (16.0%); stress (12.8%); fatigue and tiredness (11.7%) and digestive problems (10.6%). Chi et al. (1996) found the most common reasons for TCM use in Taiwan were musculoskeletal complaints (21%), injuries (15.3%) and digestive complaints (11.4%) [16]. Chen et al. (2006) reported similar reasons for TCM utilization in Taiwan [14]. Kang et al. (1998) also indicated the most common conditions treated by TCM practitioners were muscular problems (59.9%), bone fractures (35.5%), fatigue and weakness (25.8%) and respiratory conditions (25%) [17]. Different categorizations of study groups and reasons for seeking TCM in these studies limit the comparability of these findings to the results of this study. Nevertheless, participants utilized TCM for reasons akin to those of local TCM users. Almost two thirds (70.2%) of participants stated the treatment they received was effective. There is no comparative research on use of TCM by Westerners in Taiwan or elsewhere in Asia. Perspectives of effectiveness and communication between patients and health providers can be expected to vary between cultures [18]. A UK study of patient perspectives of complementary and alternative medicine (CAM) services under the National Health Services showed satisfaction with clinical care was high, with 81% indicating their problem had improved very much, moderately or slightly [19]. Singh et al. (2004) found 79% of CAM users from an Indian community living in South Africa reported positive outcomes after treatment [20].

The non-Asian workers in this study who had a family member or friend to help in use of TCM were more than twice as likely to be TCM users (OR 2.47). This result suggested that foreigners in Taiwan needed assistance in using TCM, which could be due to limitations in local language ability, lack of familiarity with TCM theories and treatment, and cultural differences. In Canada, Kelner and Wellman also found personal recommendation of a particular practitioner by a friend or family member was the main reason given by users for their choice of acupuncture and TCM [21]. The high association between very good to adequate information about TCM services in Taiwan and use of these services also suggests knowledge of TCM services affected the likelihood of utilization. A similar importance of patient knowledge of services available was also demonstrated in previous studies [13,18,20,22]. Eisenberg (1993) and Coe (1999) also reported the most common reason identified for not using CAM is a lack of knowledge about what these services provide [18,23]. Participants with an intermediate to advanced ability to read Chinese were most likely to be TCM users (OR 3.26). As an enabling factor, a high ability to read Chinese is also an indicator of acculturation i.e. knowledge of and/or experience with cultural nuances, values and practices in Taiwan. Chinese characters not only represent lexical vocabulary, but also contain symbols or radicals related to Chinese culture and tradition which affects how the characters are interpreted. Thus, the significance of this result was not unexpected. Participants with a high ability to read Mandarin were likely to have a better knowledge of TCM and better understanding and communication with TCM providers.

A significantly associated predisposing factor influencing TCM use in this study was age. This finding is consistent with Chen et al (2006), who reported that TCM utilization in Taiwan peaked in individuals around 30 years old and within the age ranges found to be significant in other findings of CAM utilization [14,21,24,25]. Australian findings also demonstrated individuals between 25–44 years old were more likely to use CAM [22].

A higher likelihood was found between participants who had visited a modern allopathic doctor about a health problem in the previous year (OR 2.67). This result is consistent with other findings in Taiwan where use of both modern allopathic medicine and TCM are available [26]. Acupuncture patients have shown a positive association with the use of other health services [27]. Other research including that from Eisenberg (2001) and the World Health Organization (2000) also suggest many CAM users seek these therapies when allopathic medical treatment is ineffective or undesirable and when a combination of both treatment methods may be superior to only one [10,28-30]. In Taiwan the high availability and affordability of both modern allopathic medicine and TCM may also enable utilization of both, but further research is required to examine whether TCM is used as a complement to or a substitute for allopathic medicine.

### Limitations

This study population can be described as healthy because these workers are required to have a health check-up including a chest x-ray and blood test within 4 months of arrival in Taiwan in order to obtain a work permit, NHIS eligibility and resident visa. This study did not apply random sampling, but according to available statistics the gender and nationalities of participants included is not significantly different from that of all white collar foreign workers living in Taiwan [3]. The establishment of causal relationships is beyond the limits of the design used in this study. The TCM therapies listed in this study correspond to the most common forms of TCM listed in local studies and research conducted by the WHO [29]. In Western countries use of TCM is often studied under the broader definition of CAM utilization, thus the comparability of the findings of this study with other study results needs to take the definition of TCM use into account.

## Conclusion

This study found the prevalence of TCM utilization by non-Asian white collar foreign workers in Taiwan was higher than that in studies in the general population reported in Western countries. Predisposing factors (age), enabling factors (ability to read Chinese, family and friends available to assist in TCM use, and information on TCM services) and need factors (visit to an allopathic medical doctor), were the factors significantly associated with TCM use. Additionally, enabling factors appear to be more influential in the utilization of TCM by this population than predisposing or need factors under the model of health service utilization used in this study. This study provides new information on the pattern of TCM use by Western workers in an Asian country. Our results suggest that Western workers are likely to try traditional medicine that is different from that in their cultural background when Western allopathic medical services are also available and affordable. Strategies could be developed to overcome enabling factor-related barriers to increase access by Westerners to TCM services.

## Competing interests

The authors declare that they have no competing interests.

## Authors' contributions

MD participated in the design of the study, collected the data, performed the statistical analysis, drafted and revised the manuscript. CT gave advice in designing and interpreting the results, and co-wrote the manuscript. CD gave advice in interpreting the results and revising the manuscript. LC supervised the whole study process, collected comments from all authors, revised and finalized the manuscript draft.

## Pre-publication history

The pre-publication history for this paper can be accessed here:


